# Altered interhemispheric functional connectivity in patients with comitant exotropia before and after surgery: a resting-state fMRI study

**DOI:** 10.3389/fnhum.2023.1095431

**Published:** 2023-07-27

**Authors:** Xiang-Xun Chen, Wen Chen, Hao Hu, Meng Zhao, Hu Liu, Xiao-Quan Xu, Fei-Yun Wu, Jie Wang

**Affiliations:** ^1^Department of Radiology, The First Affiliated Hospital of Nanjing Medical University, Nanjing, China; ^2^Department of Ophthalmology, The First Affiliated Hospital of Nanjing Medical University, Nanjing, China; ^3^Department of Interventional Radiology, The First Affiliated Hospital of Nanjing Medical University, Nanjing, China

**Keywords:** comitant exotropia, resting-state functional magnetic resonance imaging, voxel-mirrored homotopic connectivity, visual cortex, cerebellum

## Abstract

**Purpose:**

To assess the interhemispheric homotopic connectivity alterations in patients with comitant exotropia (CE) before and after surgery, using resting-state functional magnetic resonance imaging (rs-fMRI) with voxel-mirrored homotopic connectivity (VMHC).

**Methods:**

Thirty-four patients with CE and twenty-four well-matched healthy controls (HCs) were enrolled to undergo a preoperative rs-fMRI scan. The rs-fMRI scan was performed again in twenty-four patients 1 month after surgery. The VMHC method was applied to evaluate the group differences of interhemispheric functional connectivity. The correlations between VMHC values and clinical variables were analyzed in the patient group.

**Results:**

Compared with HCs, 34 patients with CE showed significantly increased VMHC values in occipital lobe (cuneus/superior occipital gyrus/middle occipital gyrus/calcarine), cerebellar area 8/cerebellar Crus1 area, and cerebellar Crus1 area. In CE group, VMHC in the cuneus was positively correlated with stereoacuity (*r* = 0.417, *P* = 0.014), meanwhile VMHC in the cerebellar Crus1 area was positively correlated with stereoacuity (*r* = 0.395, *P* = 0.021). One month after surgery, the 24 CE patients with follow-up showed decreased VMHC values in the cuneus and superior occipital gyrus compared with preoperative collection, meanwhile, non-significant difference compared with HCs.

**Conclusion:**

Our study revealed the interhemispheric homotopic connectivity changes of patients with CE in the occipital lobe and cerebellum before and after surgery. The findings may provide a new perspective for the neurological alterations of CE.

## 1. Introduction

Strabismus is a worldwide health concern with the pooled prevalence of 1.78% in individuals less than 20 years of age (Hashemi et al., 2019). Anisometropia, heritance and critical retinopathy of prematurity may be the greatest independent risk factors of strabismus (Pennefather et al., 1999; Birch et al., 2005; Maconachie et al., 2013; McKean-Cowdin et al., 2013). Comitant strabismus develops most commonly in early childhood. It is defined as an equal angle of ocular misalignment in all fields of gaze, without limitation of eye movement associated with paralytic or mechanical etiologies (Oystreck and Lyons, 2012). Comitant exotropia (CE) is a common type of strabismus characterized by ocular deviation and stereopsis dysfunction, which impacts the psychosocial health and quality of life of children and parents (Buffenn, 2021). At present, the rectus muscle surgery is the main treatment approach for CE patients. Although patients could benefit from the correction of deviations, remained difficulty in re-establishing stereopsis eye balance or recurrent poor binocularity may sometimes occur after surgery, resulting in the overall successful rate of surgery being only 60.3% (Livir-Rallatos et al., 2002; Oh and Hwang, 2006). Hence, there is a need to illuminate the underlying organic basis of the remnant stereopsis disturbance and visual imbalance, in order to potentially improve the efficiency of clinical interventions and patients’ quality of life (Chan et al., 2004).

Resting-state fMRI (rs-fMRI) has been proven to be helpful to study changes in brain neural activity in patients with CE. A previous study showed that patients with CE had increased functional connectivity between the posterior primary visual cortex and other cortical areas (Yan et al., 2019). Another study showed changed brain network activity in brain regions involved in ocular motility in patients with CE (Tan et al., 2018). These studies demonstrated that patients with CE had brain alterations in vision and eye-motor control regions. However, they only focused on preoperative brain changes of CE. The brain functional variations before and after corrective surgery in CE patients remain largely unknown. One recent study applying amplitude of low-frequency fluctuation (ALFF) method revealed the brain activity changes of patients with CE in visual-associated areas along with surgery (Wu et al., 2022). However, this preliminary study only used basic methodology and overlooked the interhemispheric functional integration of brain activity. Further researches with methods focused on functional interaction between hemispheres are needed to uncover the neural patterns before and after surgery in CEs.

As a novel data-driven method of rs-fMRI, voxel-mirrored homotopic connectivity (VMHC) quantifies the resting-state functional connectivity between individual voxel in one hemisphere and its mirrored counterpart (Zuo et al., 2010). It measures integrity of information communication between hemispheres. As a functional integration approach, VMHC assesses the brain as an integrated network, which differs from functional segregation indices (such as ALFF), may be useful to identify more abnormalities and explain the neural mechanism of diseases from a distinct perspective. Researches have demonstrated that VMHC is useful in exploring the interhemispheric functional connectivity in various neurological disorders and ophthalmic diseases, such as Parkinson’s disease, glaucoma, diabetic nephropathy complicated by retinopathy, thyroid-associated ophthalmopathy, and amblyopia (Wang et al., 2020; Chen et al., 2021; Peng et al., 2021; Tong et al., 2022; Zhang et al., 2022). A recent study also demonstrated interhemispheric homotopic connectivity alterations in the cerebellum in CE patients (Chen et al., 2022). Given the previous neuroimaging evidence and the postoperative recovery of visual function, we hypothesized that the interhemispheric functional connectivity alteration after surgery in patients with CE could also be detected by using VMHC method.

Therefore, the purpose of this study was to investigate the VMHC alterations in patients with CE before and after surgery.

## 2. Materials and methods

### 2.1. Subjects

Thirty-four patients with congenital CE (29 intermittent exotropia and 5 constant exotropia, 15 females and 19 males, mean age 11.26 ± 5.80 years) and twenty-four healthy controls (HCs; 14 females and 10 males, mean age 11.25 ± 6.26 years) were recruited from our hospital. The patients were recruited according to the following criteria: (1) binocular best-corrected visual acuity (BCVA) ≥ 1.0; (2) without history of eye surgery or other ocular diseases (amblyopia, glaucoma, cataracts, inflammation, etc.); (3) no neurological or psychiatric disorders; (4) normal brain parenchyma on cranial MRI. All patients underwent suitable surgery to correct strabismus according to the subtype and extent of exodeviation. Twenty-four of the 34 patients (9 females and 15 males, mean age 10.58 ± 3.99 years) were followed up 1 month after surgery and were enrolled in the postoperative group with CE. Dataset of 21 CE patients and 13 HCs have been used in our prior research (Wu et al., 2022), although we applied a distinct analytic approach. This study was approved by the institutional ethical review board of the First Affiliated Hospital of Nanjing Medical University. Informed consents were acquired from all the subjects.

### 2.2. Clinical assessment

All subjects received detailed ocular examinations, including visual acuity, stereoacuity and examinations of the anterior segment and the fundus. Patients with CE underwent prism deviation measurement. Visual acuity was examined through Standardized Logarithm Visual Acuity chart. Stereoacuity was measured with the Titmus test (Vision Assessment Corporation, Illinois, USA 60007) at 40 cm distance. The result was evaluated as a continuous outcome by converting seconds of arc scores to log arc/sec values as follows: 20 (1.30), 25 (1.40), 32 (1.51), 40 (1.60), 50 (1.70), 63 (1.80), 100 (2.00), 160 (2.20), 200 (2.30), 400 (2.60). The results were recorded as 3000 (3.48) when patients had no stereopsis (Seo and Kim, 2015). The angle of exodeviation was tested at near (33 cm) and distance (6 m), recorded as near and distance prism exodeviation.

### 2.3. MRI acquisition

The MRI scanning was performed on a 3.0 T MR scanner (MAGNETOM Skyra, Siemens Healthcare, Erlangen, Germany) with a 20-channel head and neck coil. The subjects were required to lie still in the supine position with eyes closed and relaxed without falling asleep. High-resolution 3D T1-weighted images were collected using magnetization prepared rapid gradient echo (MP-RAGE) sequence with the following parameters: echo time (TE) = 2.45 ms; repetition time (TR) = 1900 ms; flip angle = 9°; voxel size = 1 × 1 × 1 mm^3^; field of view (FOV) = 256 × 256 mm^2^; matrix size = 256 × 256; slice thickness = 1mm; 176 slices. Functional images were obtained by an echo planar imaging sequence. The parameters were as follows: TE = 30 ms; TR = 2000 ms; flip angle = 90°; voxel size = 3.75 mm × 3.75 mm × 4 mm; FOV = 240 × 240 mm^2^, matrix size = 64 × 64; slice thickness = 4.0 mm; 35 slices. The scanning time was 12 min and 26 s in total.

### 2.4. fMRI data preprocessing

All the rs-fMRI data were preprocessed by using Data Processing Assistant for Resting-State fMRI advanced edition (DPARSFA) V4.4 (Chao-Gan and Yu-Feng, 2010)^1^ based on SPM12 (Ashburner, 2012).^2^ Briefly, the preprocessing procedures were as follows: (1) converting Digital Imaging and Communications in Medicine (DICOM) files to Neuroimaging Informatics Technology Initiative (NIFTI) images; (2) removing the first 10 functional volumes to allow for equilibration of the magnetic field and for adaptation of the participants to the scanning environment; (3) slice timing correction for the remaining 230 fMRI images; (4) realignment for head motion correction; (5) reorientation of the structural and functional images; (6) segmentation of the structural images with the Diffeomorphic Anatomical Registration Through Exponentiated Lie Algebra (DARTEL) method (Ashburner, 2007) and generation of a group template; (7) spatial normalization to the Montreal Neurological Institute (MNI) template (resampling voxel size = 3 mm × 3 mm × 3 mm) using the segmented information from DARTEL; (8) spatial smoothing with a 6-mm full-width at half-maximum Gaussian kernel; (9) nuisance covariates regression (including the Friston 24-parameter model (Friston et al., 1996), signals of linear drift, white matter and cerebrospinal fluid); and (10) temporal band-pass filtering (frequency range of 0.01–0.08 Hz). If the maximum value of the head translation (rotation) movement was over 3.0 mm (3.0°), the whole dataset of this participant would be discarded. In our study, all the subjects were preserved after head motion correction.

### 2.5. Voxel-mirrored homotopic connectivity analysis

Voxel-mirrored homotopic connectivity computation was also performed using DPARSFA V4.4. First, a mean image was created by averaging the normalized T1-weighted images for all subjects. Second, this image was averaged with its left-right mirrored version to generate a group-specific symmetrical template. The normalized T1 images were then registered to the symmetric template and applied to the non-linear transformation to the normalized functional images. Finally, for each subject, the VMHC values were calculated as the Pearson’s correlation between the time series of each pair of mirrored interhemispheric voxels. Fisher r-to-z transformation was performed for the correlation coefficients to increase the normality of the distribution, and the VMHC z-maps were used for the subsequent analyses (Zuo et al., 2010).

### 2.6. Statistical analysis

Demographic and clinical data were analyzed using SPSS 25.0 (SPSS, Chicago, IL, USA). For continuous variables, two-sample *t*-tests (for data with normal distribution) and Mann–Whitney U tests (for data with non-normal distribution) were adopted for comparisons between the patient group and HCs. Paired sample *t*-test was applied to compare between postoperative and corresponding preoperative conditions. Chi-square tests were employed to analyze the categorical data. The statistically significant threshold was *P* < 0.05.

For the VMHC values, statistical analyses were performed using SPM12. Two sample *t*-test was used to compare the group differences between the preoperative patients and HCs, as well as between the postoperative patients and HCs. Paired sample *t*-test was conducted to examine the differences between the postoperative and corresponding preoperativeconditions. Statistical significance was based on a familywise error (FWE) correction for multiple comparisons at the cluster level (P_FWE_ < 0.05) with a cluster-defining threshold of *P* < 0.001, in line with the current reporting guideline (Eklund et al., 2016).

The mean VMHC values in each significant cluster were extracted for each subject. Spearman’s and Pearson’s correlation analyses were performed to evaluate the relationships between VMHC values and clinical parameters in preoperative patients with CE. The statistical significance threshold was set at *P* < 0.05.

## 3. Results

### 3.1. Demographic and clinical data

There were no significant differences in age (two-sample *t*-test, *P* = 0.993), gender (Chi-square test, *P* = 0.286), handedness (Chi-square test, *P* > 0.999) and bilateral BCVA (left 1.02 ± 0.06 vs. 1.05 ± 0.09, two-sample *t*-test, *P* = 0.125; right 1.01 ± 0.05 vs. 1.05 ± 0.09, two-sample *t*-test, *P* = 0.063) between 34 preoperative patients with CE and HCs. The patient group showed significantly higher scores of stereoacuity than HCs (1.88 ± 0.58 vs. 1.35 ± 0.08, two-sample *t*-test, *P* < 0.001). The mean disease duration was 4.58 ± 5.03 years for the patients. The near and distance prism exodeviation for the patients were 38.47 ± 12.35 PD and 30.81 ± 10.67 PD, respectively. Details regarding demographic and clinical data between 34 patients with CE and HCs are presented in Table 1.

**TABLE 1:** Demographic and clinical information of patients with comitant exotropia (CE) and healthy controls (HCs).

Sample characteristics	CE	HC	*P*-value
	(*n* = 34)	(*n* = 24)	
Age (years)	11.26 ± 5.80	11.25 ± 6.26	0.993
Gender (Female/Male)	15/19	14/10	0.286
Handedness	34R	24R	>0.999
Disease duration (years)	4.58 ± 5.03	–	
BCVA-L	1.02 ± 0.06	1.05 ± 0.09	0.125
BCVA-R	1.01 ± 0.05	1.05 ± 0.09	0.063
Stereoacuity	1.88 ± 0.58	1.35 ± 0.08	<0.001
Near prism exodeviation (PD)	38.47 ± 12.35	–	
Distance prism exodeviation (PD)	30.81 ± 10.67	–	

CE, comitant exotropia; HC, healthy control; BCVA, best-corrected visual acuity; L, left; R, right; PD, prism diopter.

After surgery, the 24 patients with follow-up showed significantly decreased scores of stereoacuity (1.56 ± 0.22 vs. 1.77 ± 0.54, paired sample *t*-test, *P* = 0.038) as well as lower near (11.00 ± 7.09 vs. 36.50 ± 10.24, paired sample *t*-test, *P* < 0.001) and distance (7.33 ± 6.80 vs. 28.75 ± 8.11, paired sample *t*-test, *P* < 0.001) prism exodeviation compared with corresponding preoperative clinical assessments, while still higher scores of stereoacuity than HCs (1.56 ± 0.22 vs. 1.35 ± 0.08, two-sample *t*-test, *P* < 0.001). However, no obvious differences were observed in bilateral BCVA neither between postoperative and corresponding preoperative collections for the 24 patients (left 1.03 ± 0.08 vs. 1.01 ± 0.04, paired sample *t*-test, *P* = 0.185; right 1.03 ± 0.08 vs. 1.01 ± 0.04, paired sample *t*-test, *P* = 0.185), nor between their postoperative collection and those of HCs (left 1.03 ± 0.08 vs. 1.05 ± 0.09, two-sample *t*-test, *P* = 0.488; right 1.03 ± 0.08 vs. 1.05 ± 0.09, two-sample *t*-test, *P* = 0.488). Among the 24 postoperative patients, 1 patient had diplopia while none of them had amblyopia. Details of the 24 patients with both preoperative and postoperative demographic and clinical data are presented in Table 2.

**TABLE 2:** Demographic and clinical information of postoperative CE (post-CE) and corresponding preoperative CE (pre-CE) patients as well as HCs.

Sample characteristics	Pre-CE	Post-CE	HC	^a^*P*-value	^b^*P*-value
	(*n* = 24)	(*n* = 24)	(*n* = 24)		
Age (year)	10.58 ± 3.99		11.25 ± 6.26		0.662
Gender (Female/Male)	9/15		14/10		0.149
Disease duration (years)	3.68 ± 3.74				
BCVA-L	1.01 ± 0.04	1.03 ± 0.08	1.05 ± 0.09	0.185	0.488
BCVA-R	1.01 ± 0.04	1.03 ± 0.08	1.05 ± 0.09	0.185	0.488
Stereoacuity	1.77 ± 0.54	1.56 ± 0.22	1.35 ± 0.08	0.038	<0.001
Near prism exodeviation (PD)	36.50 ± 10.24	11.00 ± 7.09	–	<0.001	
Distance prism exodeviation (PD)	28.75 ± 8.11	7.33 ± 6.80	–	<0.001	

^a^*P*-value indicated comparison between post-CE and pre-CE patients.

^b^*P*-value indicated comparison between post-CE and HCs. CE, comitant exotropia; HC, healthy control; BCVA, best-corrected visual acuity; L, left; R, right; PD, prism diopter.

### 3.2. VMHC differences

**FIGURE 1:**
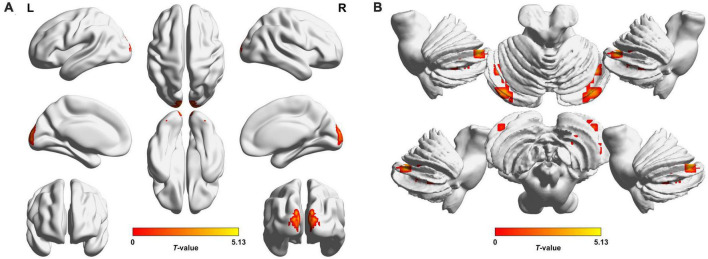
Brain regions with significant voxel-mirrored homotopic connectivity (VMHC) differences between patients with comitant exotropia (CE) and healthy controls (HCs) in the cerebrum **(A)** and cerebellum **(B)**. Compared with HCs, CE group showed significantly increased VMHC values in the CUN/SOG/MOG/CAL, Cerebelum_8/Cerebelum_Crus1, and Cerebelum_Crus1 (voxel *P* < 0.001, cluster *P* < 0.05, cluster-level FWE corrected). The warm color denotes relatively higher values in the CE group, and the color bar indicates the *T*-value from two-sample *t*-test between CE group and HCs. VMHC, voxel-mirrored homotopic connectivity; CE, comitant exotropia; HCs, healthy controls; CUN, cuneus; SOG, superior occipital gyrus; MOG, middle occipital gyrus; CAL, calcarine; Cerebelum_8, cerebellar area 8; Cerebelum_Crus1, cerebellar Crus1 area; FWE, familywise error; L, left; R, right.

**FIGURE 2:**
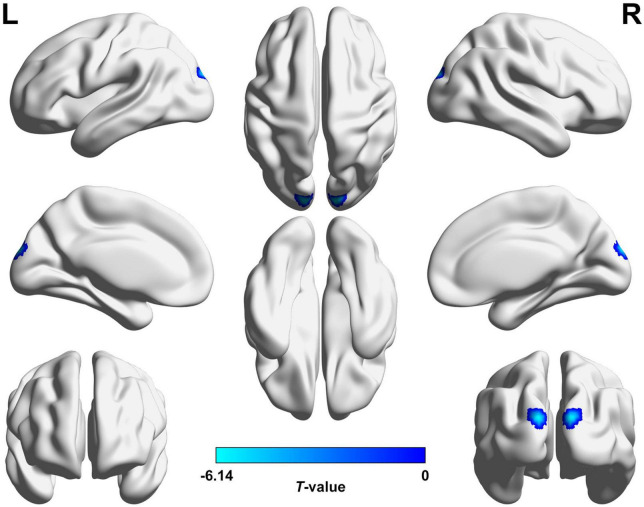
Brain regions with significant VMHC differences between postoperative and corresponding preoperative conditions. Decreased VMHC values in the CUN/SOG were observed in postoperative CE patients compared to corresponding preoperative condition (voxel *P* < 0.001, cluster *P* < 0.05, cluster-level FWE corrected). The cold color denotes relatively lower values in the postoperative condition, and the color bar indicates the *T*-value from paired *t*-test between postoperative and preoperative conditions. VMHC, voxel-mirrored homotopic connectivity; CE, comitant exotropia; CUN, cuneus; SOG, superior occipital gyrus; FWE, familywise error; L, left; R, right.

Preoperatively, the 34 patients with CE showed significantly increased VMHC values in the occipital lobe (cuneus [CUN]/superior occipital gyrus [SOG]/middle occipital gyrus [MOG]/calcarine [CAL]), cerebellar area 8/cerebellar Crus1 area, and cerebellar Crus1 area than that of HCs (Figure 1 and Table 3; voxel *P* < 0.001, cluster *P* < 0.05, cluster-level FWE corrected). Postoperatively, the 24 patients with follow-up showed significantly decreased VMHC values in one cluster located in the CUN/SOG relative to corresponding preoperative collection (Figure 2 and Table 4; voxel *P* < 0.001, cluster *P* < 0.05, cluster-level FWE corrected). In addition, there was no significant VMHC difference between postoperative group and HCs.

**TABLE 3:** Brain regions with significantly different VMHC values between patients with CE and HCs (voxel *P* < 0.001, cluster *P* < 0.05, cluster-level FWE corrected).

Brain regions/ Conditions	BA	Cluster size (number of voxels)	Peak *t*-value	Coordinates in MNI (x, y, z)
**CE group > HCs**				
R/L CUN/SOG/ MOG/CAL	17/18	44	4.910	±12, −96, 24
R/L Cerebelum_ 8/Cerebelum _Crus1	–	48	4.111	±21, −63, −39
R/L Cerebelum _Crus1	–	58	5.130	±39, −78, −24

VMHC, voxel-mirrored homotopic connectivity; CE, comitant exotropia; HCs, healthy controls; FWE, family-wise error; BA, Brodmann’s areas; MNI, Montreal Neurologic Institute; R, right; L, left; CUN, cuneus; SOG, superior occipital gyrus; MOG, middle occipital gyrus; CAL, calcarine; Cerebelum_8, cerebellar area 8; Cerebelum_Crus1, cerebellar Crus1 area.

**TABLE 4:** Brain regions with significantly different VMHC values between postoperative and corresponding preoperative conditions (voxel *P* < 0.001, cluster *P* < 0.05, cluster-level FWE corrected).

Brain regions/ Conditions	BA	Cluster size (number of voxels)	Peak *t*-value	Coordinates in MNI (x, y, z)
**Postoperative CE < preoperative CE**				
R/L CUN/SOG	18	29	−6.137	±12, −93, 24

VMHC, voxel-mirrored homotopic connectivity; CE, comitant exotropia; FWE, family-wise error; BA, Brodmann’s areas; MNI, Montreal Neurologic Institute; R, right; L, left; CUN, cuneus; SOG, superior occipital gyrus.

### 3.3. Correlation analysis

**FIGURE 3:**
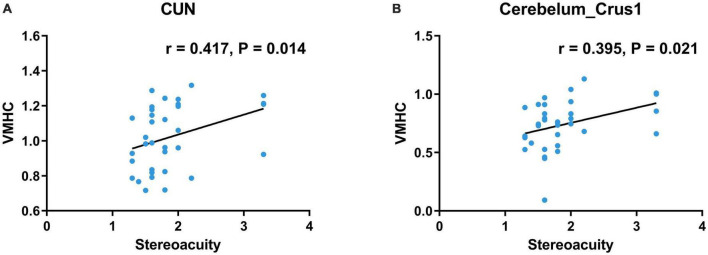
Correlations between clinical characteristics and the mean VMHC values in significant brain regions of preoperative patients with CE. **(A)** VMHC in the CUN was positively correlated with stereoacuity (*r* = 0.417, *P* = 0.014). **(B)** VMHC in the Cerebelum_Crus1 was positively correlated with stereoacuity (*r* = 0.395, *P* = 0.021). VMHC, voxel-mirrored homotopic connectivity; CE, comitant exotropia; CUN, cuneus; Cerebelum_Crus1, cerebellar Crus1 area.

In preoperative patients with CE, VMHC value in the CUN was positively correlated with stereoacuity (*r* = 0.417, *P* = 0.014, Figure 3A). Moreover, VMHC value in the cerebellar Crus1 area was positively correlated with stereoacuity (*r* = 0.395, *P* = 0.021, Figure 3B).

## 4. Discussion

We explored the interhemispheric homotopic connectivity alterations in patients with CE before and after surgery by VMHC. Our study had three main findings. First, compared with HCs, the preoperative patients with CE exhibited increased VMHC values in the occipital lobe (CUN/SOG/MOG/CAL), cerebellar Crus1 area and cerebellar area 8/cerebellar Crus1 area. Second, VMHC values in both the CUN and cerebellar Crus1 area were positively correlated with stereoacuity in preoperative patients with CE. Third, 1 month after surgery, the patients showed decreased VMHC values in occipital lobe (CUN/SOG) compared with preoperative collection, and there was no significant difference between postoperative patients and HCs.

The occipital cortex is well known to be associated with visual processing, mainly including visual formation and visual perception activities (Yu et al., 2017). Chan et al. (2004) observed that strabismus patients demonstrated decreased gray matter volume bilaterally at functional areas of V1 including the calcarine sulcus and the occipital pole. Yan et al. (2019) showed that patients with CE had increased functional connectivity between the posterior part of visual cortex and other cortical areas, which was considered to be related to stereopsis impairment. Another study demonstrated that CE patients had abnormal large-scale brain networks associated with stereoscopic vision dysfunction (Jin et al., 2022). Similarly, interhemispheric functional changes in visual cortex (CUN/SOG/MOG/CAL) were found in our study. Combined with the positive correlation between VMHC in the CUN and stereoacuity, we deduced that the observed alterations might also reflect the impaired stereopsis in CE patients.

As we know, the cerebellum participates in the execution of accurate eye movements (Herzfeld et al., 2015), while sensorimotor function is closely associated with the formation of stereovision (Ringach et al., 1996). Moreover, Gulyas and Roland observed that a number of cerebellar fields were activated in the stereopsis tasks, indicating that the cerebellum plays an important role in stereopsis mechanism (Gulyas and Roland, 1994). Considering the clinical presentation of CE patients, we deduce that the observed increased VMHC in the cerebellum might be involved in the pathogenesis of impaired stereoacuity. In addition, functional reorganization within the cerebellum in children and teenagers has been reported in previous literature (Nirkko et al., 1997; Niimura et al., 1999; Lidzba et al., 2008), implying that functional remodeling of the cerebellum after impairment is possible. Taken together, it is suggested that cerebellum area might occur functional reorganization connected with the stereopsis impairment in CE patients.

Another important finding in our study was the decrease of VMHC values in CUN/SOG after surgery. Previously, Li et al. (2017) demonstrated that patients with primary angle-closure glaucoma had reduced intrinsic functional connectivity between V1 and the attention as well as control network postoperatively. Our investigation on CE showed a similar phenomenon, i.e., decreased functional connectivity following therapy. Together with the finding that there was no significant VMHC difference between postoperative patients and HCs, the postoperatively decreased VMHC values in CUN/SOG might also indicate the functional restoration of visual cortex.

Basically, functional segregation and functional integration are the two important principles of the rs-fMRI analytic approaches (Lv et al., 2018). In our previous research using ALFF (Wu et al., 2022), we observed functional segregation abnormalities of CE patients. Extending to the prior work, the present study using VMHC further revealed that not only functional segregation, but also functional integration was disturbed in these patients. More importantly, the identified brain regions in this study partially overlapped with those in the prior research, i.e., the visual cortices were identified by both functional segregation and integration, while the cerebellar areas were only identified by functional integration. Therefore, the two principles delineate the neuroimaging properties from distinct perspectives and provide complementary information, thus combination of these metrics could more integrally characterize the brain alterations of diseases.

Our study provided relevant interpretations to the interhemispheric brain activity changes of CE, the brain activity variations along with the corrective surgery, and the postoperatively remnant stereopsis impairment. This initial study observed the increased VMHC changes in the occipital and cerebellar cortex, as well as the reversible recovery of functional alteration after treatment. Moreover, VMHC method could be available for visualization of interhemispheric functional changes in patients with CE and monitoring alterations following therapy, which could provide additional instructive value for clinicians beyond routine ophthalmic examination, subsequently improving patients’ overall quality of life.

The present study had several limitations. First, our study had a relatively small sample size and included an unbalanced number of subjects in the CE group (29 intermittent exotropia and 5 constant exotropia), which may lead to the possibility of potential biases. The small sample size (especially the constant exotropia cohort) caused the inability to conduct subgroup analyses to validate this issue. Second, the human brain is not symmetrical in general. Although we tried to solve this issue by registering the functional images to a group-specific symmetrical template to improve the functional correspondence between homotopic regions, the influence of morphometric asymmetry on the results could not be completely overlooked as well. Last, the duration of follow-up was relatively short in our study, and only a proportion of the cortices showing increased VMHC preoperatively demonstrated decreased value, representing the functional restoration after surgery. Future long-term follow-up studies are needed to observe the full-time course of functional restoration and its relationship with clinical variables.

In conclusion, our findings indicate that CE may lead to increased interhemispheric functional activities in visual-associated areas, and the brain function could partially restore along with recovery of exodeviation at 1 month after strabismus surgery. The findings may provide insight into the neurological alterations of CE.

## Data availability statement

The raw data supporting the conclusions of this article will be made available by the authors, without undue reservation.

## Ethics statement

The studies involving human participants were reviewed and approved by the Institutional Ethical Review Board of the First Affiliated Hospital of Nanjing Medical University. Written informed consent to participate in this study was provided by the participants’ legal guardian/next of kin.

## Author contributions

HH, JW, and F-YW conceptualized and designed the study. X-XC and MZ performed the MR scan. WC performed the MR data analyses. X-QX and HL contributed to the diagnosis and clinical data collection. X-XC wrote the first draft. HH provided the critical revisions of the draft. All authors approved the manuscript for submission.

## Footnotes

1
http://rfmri.org/DPARSF

2
http://www.fil.ion.ucl.ac.uk/spm/software/spm12
